# Base modifications reshape RNA folding landscapes and structure–function relationships in synthetic and natural RNAs

**DOI:** 10.21203/rs.3.rs-6465433/v1

**Published:** 2025-05-29

**Authors:** Ewan McRae, Deepak Yadav, Haoyun Yang, Sukyeong Lee

**Affiliations:** Houston Methodist Research Institute; Houston Methodist Research Institute; Houston Methodist Research Institute; Baylor College of Medicine

## Abstract

Modified bases such as 5-methylcytosine (5mC) and N1-methyl-pseudouridine (N1Ψ) are widely used to reduce the immunogenicity of and enhance the stability and translational efficiency of therapeutic RNAs, yet their effects on RNA 3D structure remain poorly understood. Here, we investigate how these base modifications influence the folding and function of structured RNAs using both an engineered RNA origami nanostructure and a naturally occurring ribozyme. We show that modified bases impair kinetic maturation of RNA nanostructures and promote alternative folding topologies via stabilization of noncanonical stacking arrangements. Cryo-EM and FRET experiments reveal that this conformational shift is driven not by changes in base pairing, but by altered stacking energetics at key junctions. Our findings highlight the need to consider structural consequences when using base modifications and offer design principles for the development of functional, structured RNAs in synthetic biology and therapeutic applications.

## Main

Modified nucleotide bases expand the chemical diversity of RNA beyond the standard four nucleotides. Naturally occurring modifications, such as pseudouridine (ψ)^1^, 2’-O-methylated nucleotides^2^, N6-methyladenosine (m6A)^3^, and 5-methylcytosine (5mC)^4^, play essential roles in RNA stability, folding, and function^5^. In therapeutic applications, modified nucleotides are used to reduce immunogenicity and enhance RNA stability and translational efficiency^6,7^. Foreign RNA can activate pattern recognition receptors such as Toll-like receptors (TLRs) and RIG-I-like receptors, triggering inflammatory responses that limit efficacy^8,9^. Modifications like N1-methyl-pseudouridine and 5-methylcytidine reduce immune recognition, enhance translation, and are used in applications such as mRNA vaccines.

However, the effects of these modified bases on RNA structure have not yet been carefully examined. While chemical probing methods can determine when base pairing patterns change between samples with different modifications, as is often the case for mRNA with many energetically equivalent alternate base pairings^10^, they fail in cases where the base pairing pattern remains the same but tertiary RNA structure has changed. RNA structure plays a critical role in regulating its function, influencing processes such as translation, stability, and subcellular localization. Utilizing these structural elements in mRNA therapies is an enticing opportunity that is currently confounded by the unknown effects of introducing base modifications to these structures.

One example of this is in the production of circular RNA therapeutics, which frequently rely on group I intron RNA enzymes (ribozymes) for self-splicing and circularization^11^. These ribozymes, which have been repurposed to create circular RNAs via permuted intron-exon (PIE) strategies^12^, require precise tertiary structures to maintain catalytic activity and are currently restricted to using nonmodified nucleotides. Given that these enzymes derive their function from their three-dimensional conformations^13–15^, introducing modified bases may disrupt their folding and impair function, posing a barrier to their application in circular RNA therapeutics.

Recent advances in synthetic biology have further expanded the potential of RNA as a programmable material. RNA origami, which employs the principles of RNA folding and modular assembly^16^, enables the design of complex RNA architectures^17,18^. These structures hold promises for applications in synthetic mRNA regulation and aptamer-based RNA therapeutics. Many different scaffolded RNA architectures^19–21^ can be used to position functional domains that regulate transcription^22^ and translation^23^ or protein antagonism^24^ via steric hindrance or structural remodeling. Furthermore, targeted delivery of therapeutic payloads with high specificity mediated by strand displacement mechanisms could be the next frontier in RNA medicine^25–27^. Incorporating modified nucleotide bases into these synthetic constructs offers additional opportunities to fine tune their stability, immune compatibility, and functional properties.

This study explores the interplay between modified nucleotide bases and RNA structure. To our knowledge these are the first structures determined for RNA transcribed constitutively with modified RNA bases. By examining both natural and synthetic RNA systems, we aim to uncover principles that can inform the design of next generation RNA therapeutics and nanomaterials.

### 5mC and N1Ψ affect RNA origami maturation and tertiary structure

In previous work we designed a 6-helix bundle of RNA with a clasp helix (6HBC)^18,28^ and showed that it cotranscriptionally folds into a kinetically trapped conformation with the clasp helix at a ~ 45 degree angle with respect to helices 1–5. A slow maturation process, which requires the breaking and reformation of a 6 base pair kissing loop, has been studied by small angle x-ray scattering and confirmed by cryo-EM reconstructions revealing a final conformation where the clasp helix is approximately parallel with helices 1–5.

To investigate whether modified nucleobases could affect this maturation process we transcribed 6HBC with either a complete replacement of cytosine with 5-methyl cytosine (5mC) or uridine with N1-methyl-pseudouridine (N1Ψ). Prior to plunge freezing and subsequent cryo-EM imaging samples were allowed 34 hours to mature, twice the time required.

**Figure 1:**
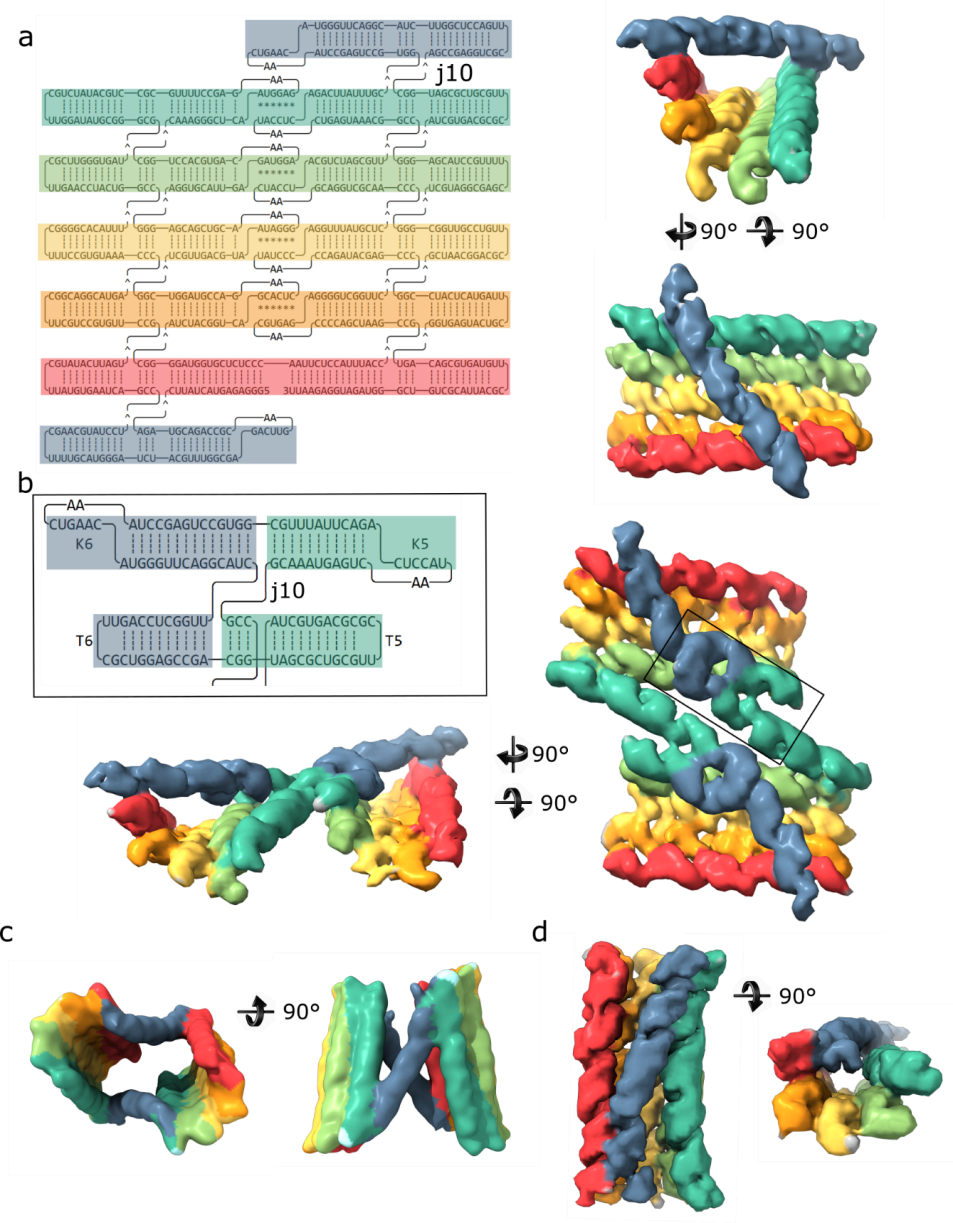
(a) 2D structure (left) and cryo-EM reconstruction (right) of the monomeric 5mC 6HBC RNA. (b) Zoom in view of the 2D structure of the misfolded junction in the type-1 6HBC dimer (full blueprint in Supplemental figure 9) and cryo-EM reconstruction of the type 1 dimer from the 5mC 6HBC dataset. (c) Cryo-EM reconstruction of the type 2 dimer from the 5mC 6HBC dataset. (d) Cryo-EM reconstruction of the monomeric 5mC 6HBC-TC RNA.

For the 5mC 6HBC sample, single particle averaging methods could not identify any particles in the mature conformation but did result in a class resembling the early conformation (Fig. 1a & Supplemental Fig. 1). Despite our best efforts we were unable to reconstruct a monomeric N1Ψ 6HBC from our data. However, for both the 5mC & N1Ψ 6HBC samples we observed classes that resemble dimeric structures, which have previously not observed with nonmodified nucleotides. Two distinct classes of dimeric structure were observed.

The type-1 dimer (Fig. 1b & Supplemental Fig. 2&3) was most abundant in the 5mC-6HBC dataset. Each 6HBC contains 10, 4-way, junctions that we designed to form stacking arrangements to minimize backbone torsional strain and promote the internal pseudoknot formation. Type-1 dimer appears to have an alternate arrangement of junction 10, which connects the K5 helix, the K6 helix, the T6 hairpin and the T5 hairpin. Whereas our design has K5 stacking with T5 and K6 stacking with T6 (Fig. 1a), type-1 dimer has the stacking arrangement of K5 with K6 and T6 with T5 (Fig. 1b Supplemental Fig. 9). In this arrangement K6 can still form the intramolecular ‘clasp’ pseudoknot with K6’ and K5 is oriented away from the bundle allowing for the formation of two intermolecular K5-K5’ Pseudo-knots. T6 and T5 also coaxially stack, forming a short 2 component helical element with T6 interacting with the major groove of the helix preceding K6. More abundant in the N1Ψ dataset, The type-2 dimer (Fig. 1c & Supplemental Figs. 4 & 6) is connected by two intermolecular K6-K6’ pseudo-knots.

### Twist correction affects maturation of 6HBC but not dimerization

Our previous work^18^ identified a twist correction that can be applied to our RNA origami architecture that amounts to a 1bp difference in the ideal integer number of helical turns between double crossover motifs and reduces the twist defect observed in our structures. By adding a single base pair to our designs, before each KL, we corrected the twist in a 5-helix tile design but had not applied it to our 6HB designs. Here we have applied the twist correction (TC) to our 6HBC template (Supplemental Fig. 10) and investigated its effect on the modified base 6HBC-TC. To our surprise, this resulted in the monomeric state of the 5mC 6HBC-TC resembling the mature conformation even when grids were prepared immediately post purification (Fig. 1d & Supplemental Fig. 5). However, we were still unable to reconstruct any monomeric N1Ψ 6HBC-TC, observing only dimeric structures in our cryo-EM data set (Supplemental Figs. 3 & 4).

### Alternate stacking arrangements can be mitigated by avoiding modified bases at junctions

The alternate stacking arrangement in type-1 dimer is such that the helices are stacked in the order in which they are transcribed. KL5 is transcribed first followed by KL6 and then H6 and then H5, thus KL5 can first stack with KL6, presumably, before H5 is even transcribed. We hypothesized that the reason for the appearance of the type-1 dimer in the base-modified 6HBC samples and not the nonmodified 6HBC sample was due to the stabilization of this alternate stacking arrangement during cotranscriptional folding when modified bases are present at the junction. The extra stacking energy from the modified bases could shift the equilibrium of junction conformations, allowing the alternate stack to persist for long enough to form the intermolecular PKs and lock the structure in this alternate state.

To test this, we altered the sequence of the bases at junction 10 of 6HBC-TC (Supplemental Fig. 11) such that there are no cytosines at the junction.

**Figure 2:**
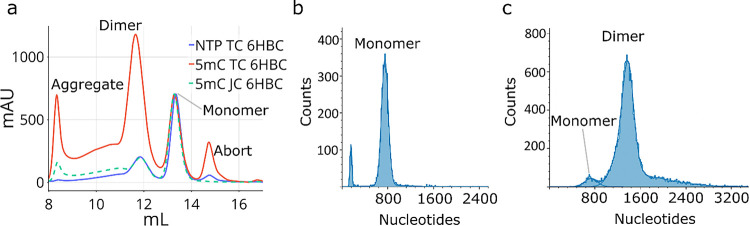
(a) SEC trace of the 5mC 6HBC-TC, NTP 6HBC-TC and 5mC 6HBC-JC RNA normalized to the monomer peak height. (b) Mass photometry data from fractions collected from the monomer peak of the 5mC 6HBC-TC trace in (a). (c) Mass photometry data from fractions collected from the monomer peak of the 5mC 6HBC-JC trace in (a).

To better quantify dimer formation in our 6HBC samples we performed analytical size exclusion chromatography (SEC) on a Superose 6 increase column (3.2/300mm) to resolve the monomer and dimer peaks. Purifying our transcription reactions by this method revealed an aggregate peak, a dimer peak, a monomer peak, an aborted product peak and the NTP peak (Fig. 2a). We confirmed the identity of the monomer and dimer peaks with mass photometry on a 2MP instrument, which gave size estimates of ~ 750nt and ~ 1500nt, respectively (Fig. 2b,c). The dimer sample appeared to contain a portion of monomeric particle (~5%), but this did not increase as we diluted the sample down to 2.5nM concentration and we attribute it to peak overlap caused by diffusion during our fractionation (Figure S8a). The monomer sample gave a consistent size estimate across all concentrations tested, indicating that the monomer and dimer populations are determined cotranscriptionally and are not concentration dependent after their initial folding (Figure S8b).

When we compare the SEC traces between the 5mC-6HBC-TC and NTP-6HBC-TC samples we see that the dimer peak is more abundant in the 5mC sample but is not absent in the NTP sample as we had anticipated (Fig. 2a). Transcribing and purifying the 5mC 6HBC-JC (junction corrected) sample results in a trace that is nearly identical to the NTP 6HBC-TC trace (Fig. 2a), indicating that the junction correction has substantially reduced the formation of dimeric 6HBC. We propose that the remainder of dimeric 6HBC in the 5MC 6HBC-JC sample as well as the dimeric peak in the 6HBC NTP sample are type-2 and that type-2 dimer formation could be dependent on the transcription initiation rate and local concentration of partially folded 6HBC when the clasp helix has yet to form intramolecularly, which could differ from our previous study.

### The tetrahymena ribozyme has altered activity with 5mC and N1Ψ replacements

The tetrahymena group I intron ribozyme (TR) is a well-studied RNA enzyme with a well-defined structure and a misfolding pathway similar in nature to the 6HBC sample. An established protocol for TR folding^29^ is a denaturing purification and thermal refolding yielding ~ 90% of the RNA in a topological misfold. The misfolded state (M) is enzymatically inactive but can be refolded by incubation at 50°C for 30 minutes into the native, active, state (N)^30^. Recently, the 3D structures of the misfold and active form have been resolved by cryo-EM^31,32^, the maturation from inactive (M) to active (N) state necessitates the breaking of a 7 base pair (P3) helix.

To investigate how replacing cytosine and uridine with 5mC and N1Ψ would affect the folding and activity of this naturally occurring RNA structure. We chose to study the 408nt L21, Scal fragment of the ribozyme, which is missing the first 20 nucleotides and ends at the naturally occurring Scal restriction enzyme site. This version contains the internal guide sequence that facilitates substrate binding and allows for TR activity assays to be performed in trans with a separate substrate strand.

**Figure 3:**
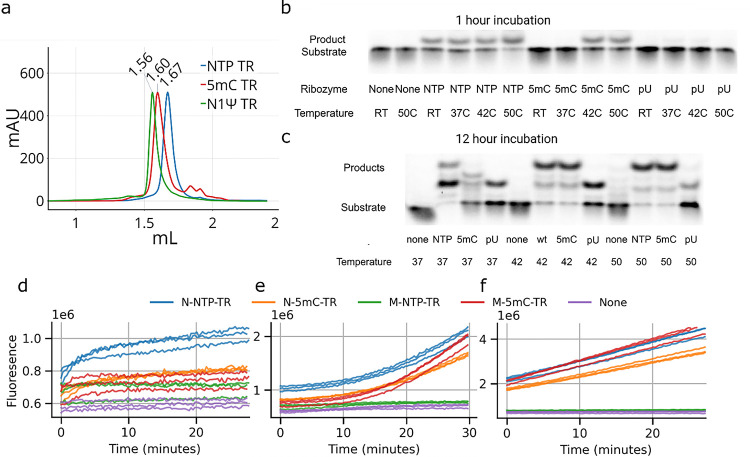
(a) SEC trace of purified NTP-TR (blue), 5mC-TR (red) and N1Ψ-TR (green). TR activity assays with Cy5-labelled substrate after 1 hour (b) and 12 hours (c). TR activity assays with FAM-BHQ labelled substrate at 25 °C (d), during heating to 42 °C (e) and at 42 °C (f).

SEC traces of what should be the M state TR revealed a slight, but consistent, difference in the elution of the profile where M-N1Ψ-TR eluted first, followed by M-5mC-TR and then M-NTP-TR, indicating the M-NTP-TR is the most compact structure (Fig. 3a).

Activity assays performed with the substrate in tenfold excess to the ribozyme were performed, Initially, for 1 hour at room temperature (25°C), 37°C, 42°C and 50°C with the three TR versions that had been ‘activated’ by refolding at 50°C for 30 minutes (Fig. 3b). These assays showed that N-NTP-TR had consistent levels of activity across the temperatures tested after 1 hour. N-5mC-TR did not produce any cleaved substrate after 1 hour at 25°C or 37°C but did produce product at 42°C and 50°C. N-N1Ψ-TR did not produce any cleavage products at 25°C or 50°C but had faintly discernible product band at 37°C and 42°C after the 1 hour incubation.

Reactions performed for 12 hours revealed the formation of a second and third, slower migrating, product (Fig. 3c). At 37°C the N-NTP-TR had converted nearly all the substrate to the first product and a smaller amount to the third product. N-5mC-TR had the least activity at 37°C, converting some of the substrate to the first product and some to an intermediate, second, product, but none to the third product. At 42°C and 50°C, N-NTP-TR and N-5mC-TR had similar activities and had processed nearly all the substrate to the third product with trace amounts of substrate, first and second product visible on the gel. N-N1Ψ-TR converted the least substrate at all temperatures and only produced the first product, with the most N-N1Ψ-TR activity observed at 42°C.

To better understand the kinetics of the reaction we ordered a separate substrate with fluorophore and quencher that allowed us to monitor the first cleavage reaction with better time resolution using a plate reader. In these experiments we followed the activity of both the misfolded ‘M’ state and refolded ‘N’ state of the NTP-TR and 5mC-TR. By the first read the 5mC-TR samples consistently had more signal than the substrate only sample and the M-NTP-TR samples indicating some amount of immediate activity but no further increase in signal after 30 minutes at room temperature, indicating that a single turnover event had happened but that the substrate was not being recycled (Fig. 3d). The N-NTP-TR sample had greater initial increase than 5mC-TR followed by a consistently increasing signal for 30 minutes at room temperature. The temperature inside the plate reader was then set to 42°C and we continued to monitor the fluorescence intensity from the samples (Fig. 3e). At an internal plate reader temperature of 42°C the N-5mC-TR, M-5mC-TR and N-NTP-TR samples showed a rapid increase in fluorescence, whereas the substrate only sample and M-NTP-TR showed only a slight increase. The increase in fluorescence continued for the 5mC-TR and N-NTP TR samples for 30 minutes with the 5mC-TR samples catching up to the N-NTP-TR sample’s fluorescence intensity levels (Fig. 3f).

### 5mC TR forms an alternate tertiary structure to NTP-TR

**Figure 4:**
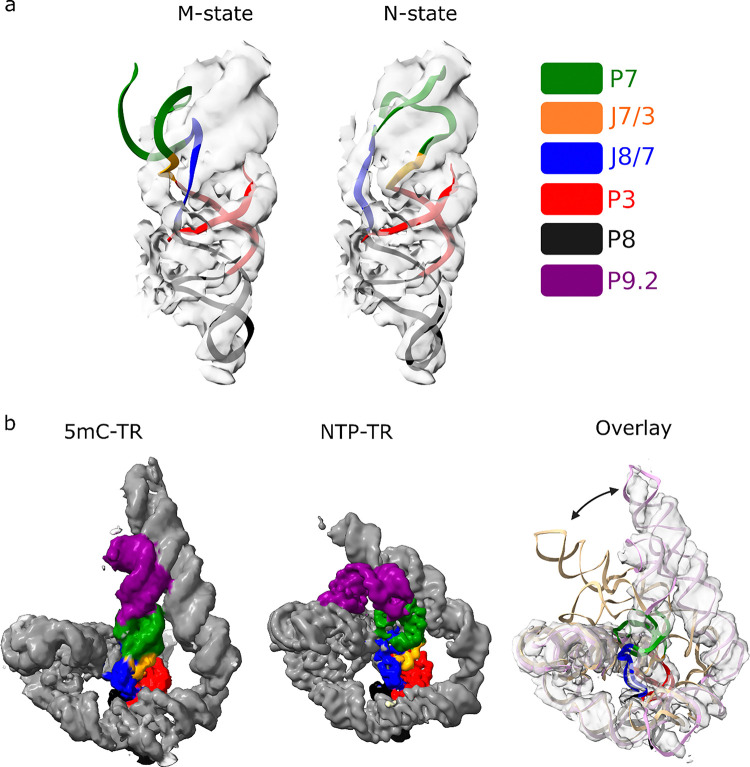
(a) Rigid body fitting of the M state model of TR (PDB:7XSM) (left) and N state model of TR (PDB:7XSN) (right) into the 5mC-TR cryo-EM map. (b) Comparison of the 5mC-TR cryo-EM map to the NTP-TR map (EMD-33428).

The FPLC purified M-5mC-TR sample was used for cryo-EM imaging and single particle analysis. After 2 rounds of sorting in 3D we were left with ~ 233,000 particles that were used to reconstruct a map to gold-standard Fourier shell correlation of ~ 5 Angstrom (0.143 cutoff). Major and minor grooves are readily apparent at this resolution, allowing for rigid body fitting of all the helical components of the TR structure. Resolution at the core of the structure is sufficient to observe J8/7 and docking the M and N state models from Li et al. into our map shows that the M-5mC-TR structure most likely adopts the N state topology, despite not undergoing the refolding process at 50°C and explaining its enzymatic activity without the refolding (Fig. 4a).

The major difference between our M-5mC-TR and previously published NTP-TR structures is the placement of the P9 and P9.1/P9.2 helices (Fig. 4b). In the M-5mC-TR structure, L9 is not positioned to make tertiary contacts with the P4/6 domain, instead, P9 is coaxially stacked on P7 and parallel to P9.2. This alters the angle of the P8,P3,P7,P9 stack with respect to the P4/P6 domain, resulting in a more solvent accessible guanosine binding pocket.

**Figure 5:**
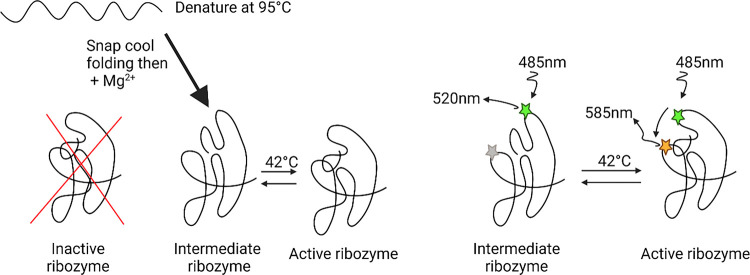
(a) Schematic depicting the proposed folding pathway of 5mC-TR and the dual-fluorophore labelling strategy for FRET experiments (b).

We propose that a dynamic equilibrium between the tertiary interaction of P5 and L9 (closed) and the coaxial stacking of P9 on P7 (open) exists. While the open conformation lacks the two adenine stack between the P9.2 loop and a bulged adenine in the P4/P6 domain it gains two stacked cytosine bases. In the 5mC-TR molecule the increased energy of base stacking from the 5-methyl group could shift the equilibrium distribution from closed to open. We propose a model where 5mC-TR can avoid the kinetically trapped inactive conformation and form an intermediate, open, state (Fig. 5a). In this open state the 5mC-TR might have low levels of activity, but when the temperature is increased to 42°C it can overcome this energy barrier and sample the closed state (Fig. 5b).

### TR P9/P7 stack exists in a dynamic equilibrium

To test the idea that the 5mC-TR can sample the closed state and NTP-TR can sample the open state we devised a FRET experiment to probe the two conformations through ensemble measurements. We used the Tiled RNA Acylation at Induced Loops (TRAIL) method to add an acceptor fluorophore to position A125 in the P4/P6 domain and a donor fluorophore in L9 at A325. Our model indicates that the 2’OH in these positions should face the solvent and their modification should not interfere with the overall structure of the RNA. Furthermore, the distance between A125 and A325 changes from 50 Angstrom to 13 Angstrom in the open and closed states, respectively, making them amenable to discrimination by FRET measurements.

**Figure 6:**
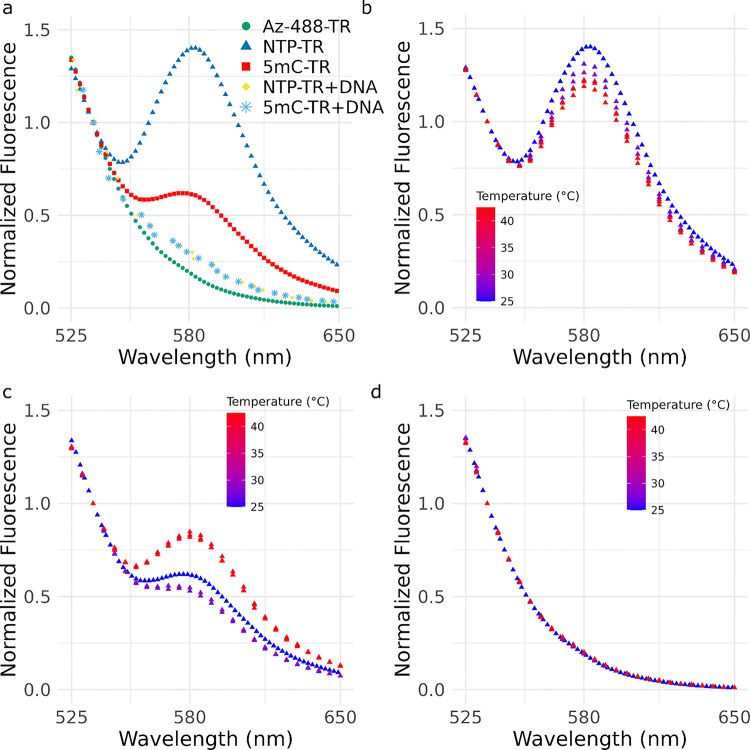
(a) Emission spectra after excitation at 485nm for the singly labelled Az-488-TR (green), NTP-TR (blue), 5mC-TR (red), NTP-TR + DNA oligoes (yellow) and 5mC-TR + DNA oligoes (light-blue). Emission spectra after excitation at 485nm measured after heating and cooling cycles for NTP-TR (b), 5mC-TR (c) and Az-488-TR (d), ambient temperature at each reading is indicated by color coding.

The fluorescence spectrum from 525–650nm was measured for 5mC-TR & NTP-TR after exciting at 485nm (Fig. 6a). The Az-488 control RNA showed minimal signal at the acceptor emission, 580nm. The NTP-TR showed fluorescence at 585nm, equivalent to 1.4 x the signal at 535nm. The 5mC-TR samples showed less fluorescence compared to NTP-TR at 585nm, only 0.6 x the signal at 535nm. When each sample was re-annealed to the DNA oligonucleotides used for the site selective acylation protocol, effectively converting the entire sequence to a double stranded RNA:DNA hybrid, minimal fluorescence was detected at 585nm. These results are consistent with our experimental design where the open state (5mC-TR) should have lower FRET efficiency than the closed state (NTP-TR) due to the larger distance between labelled nucleotides.

Samples were subjected to three heating and cooling cycles with 30 minute equilibration at 42 degrees and measurement of the emission spectra followed by 30 minute equilibration at room temperature and measurement of the emission spectra. Opposite effects were observed when the heated NTP-TR (Fig. 6b) and 5mC-TR (Fig. 6c) were measured. No effect was observed for the Az-488 control (Fig. 6d). The 5mC-TR had an increase in emission at 585nm at higher temperatures, indicating that the bases A125 and A325 were on average in closer proximity to one another. The NTP-TR sample had decreased emission at 585nm at higher temperatures, indicating that A125 and A325 were on average further away from one another. This effect appears to be at least partially reversible; both the 5mC-TR and NTP-TR sample returned to a slightly lower FRET efficiency state at room temperature, compared to their initial measurements at room temperature, after the three heating cycles.

## Conclusions

This study reveals how the incorporation of modified nucleotides, specifically 5-methylcytosine (5mC) and N1-methyl-pseudouridine (N1Ψ), reshapes the folding landscape and activity of RNA nanostructures and catalytic RNAs. Our findings support the view that RNA modifications can act as fine-tuners of structure-function relationships by altering tertiary structure equilibria. Furthermore, base stacking energetics, rather than changes in base-pairing, can play dominant roles in determining RNA tertiary topology.

Maturation of the 6HBC RNA origami structure is impeded by the presence of 5mC and both 5mC and N1Ψ modifications can lead to alternate coaxial stacking at a key junction (junction 10), which promotes intermolecular pseudoknot formation and locking of the dimer. Notably, the same misfolding of J10 has been observed in a concurrent study on the effects of 2’F modified pyrimidines. Correcting the junction sequence to remove modified bases and reduce stacking potential largely abrogated type-1 dimer formation, suggesting a strategy to avoid undesired multimerization when designing modified RNA architectures. This finding may be broadly applicable to synthetic biology efforts involving structured RNAs made with modified bases, including RNA origami, riboswitches, and therapeutic circRNAs.

Parallel studies with the Tetrahymena ribozyme (TR) reveal that 5mC and N1Ψ modifications also modulate activity through structural alterations. The modified versions of TR exhibit a temperature-sensitive activity, which differ from that of the unmodified enzyme, with 5mC-TR being mostly inactive at low temperatures but regaining function at elevated temperatures. Cryo-EM reconstruction of M-5mC-TR reveals that it does not form the topological misfold observed with M-NTP-TR. Rather, the differences in enzymatic activity are explained by the P9 domain being coaxially stacked with P7, disrupting canonical tertiary contacts with P5 and expanding the guanosine-binding pocket. FRET studies confirmed that this alternate arrangement corresponds to a more open conformation with lower FRET efficiency, which can interconvert with the closed conformation at elevated temperatures.

These observations point to a dynamic equilibrium between open and closed conformations of TR, modulated by temperature and influenced by 5mC-mediated stabilization of the open (inactive) form, suggesting that enhanced stacking energy from the methyl group may shift the conformational ensemble.

A crystal structure of a 247nt fragment of the TR shows one chain within the asymmetric crystal unit adopts this P9/P7 stack^14^, but was presumed to be a crystal packing artifact. Interestingly, Li et al. propose in their manuscript that disruption of L9-P5 interaction may be an intermediate along the maturation pathway from M to N state. Our data suggest that the open conformation is sampled by the natural ribozyme and could be important for cycling of guanosine and catalytic activity. Hinting that structures with modified bases could be used as a tool to reveal/stabilize low abundance alternate conformations of RNA.

Our work highlights a critical consideration for the use of modified nucleotides in synthetic and therapeutic RNA design. While 5mC and N1Ψ are often employed to improve stability and reduce immunogenicity, their influence on RNA folding and tertiary architecture can produce unintended consequences, including misfolding or reduced activity. This necessitates a careful evaluation of structural context, particularly in systems with intricate tertiary interactions such as ribozymes or RNA nanostructures. Future work should explore how broader classes of base modifications affect folding landscapes across diverse RNA motifs and develop guidelines or design rules to accommodate or counteract these effects. Moreover, kinetic folding models that include modified base energetics will be instrumental in advancing the predictive design of structured therapeutic RNAs and synthetic biological systems.

## Methods

### RNA transcription

DNA templates were ordered from Twist Biosciences with T7 promoter and restriction enzyme sites for linearization, cloned into a pUC19 based plasmid, purified by mini-prep (Qiagen) and linearized with either Bsal (6HBC) or Scal (TR). RNA was transcribed with Hi-Scribe T7 polymerase (NEB) substituting the manufacturer’s buffer for our own with (4mM GTP, 4mM ATP, 4mM UTP (or N1ΨUTP), 4mM CTP (or 5mCTP), 12mM MgCl2, 40mM HEPES pH 8.0, 2.5mM spermidine, 0.005% Triton X-100, 10mM DTT), 1ug of template was used per 50uL of reaction. Transcriptions were incubated for 3 hours at 37°C before 1 hour of DNase I treatment (NEB). All plasmids were sequenced by Plasmidsaurus.

### Purification of 6HBC

Samples were centrifuged for 5 minutes at 21,000g prior to loading on a Superose 6 increase (3.2×300 or 10×300) and eluted with 25mM HEPES pH 8.0, 50mM KCl, 5mM MgCl2, samples eluted as a single peak on the 10×300 column used for cryo-EM grid preparation. Samples were spin concentrated in a 0.5mL Amicon spin filter at 5000g (25C) to a final concentration of 2–2.5mg/mL.

### Purification and folding of TR

TR samples were centrifuged for 5 minutes at 21,000g prior to loading on a Superose 6 increase (3.2×300 or 10×300) and eluted with 50mM HEPES pH 8.0. The major peak was refolded by heating to 90C for 3 minutes and cooled at 25C for 15 minutes, followed by the addition of MgCl2 to a final concentration of 10mM to achieve the “M-state”. N-TR was further incubated for 30 minutes at 50C. For cryo-EM, these M and N state TR were then further purified by SEC on a Superose 6 increase equilibrated with 50 mM HEPES pH 8.0 and 10mM MgCl2. Samples were spin concentrated in an 0.5mL Amicon spin filter at 5000g (25C) to a final concentration of 2–2.5mg/mL and aliquots were made and stored at −20C.

### Cryo-EM data collection

Quantifoil 2/1 carbon film copper mesh (300) grids were glow discharged for 30 seconds at 15mA using a PELCO EasiGlow and used immediately after. Grids were prepared using a Vitrobot Mark IV, 3uL of sample as applied to the carbon foil side, blotted for 4 seconds in 100% humidity at 25C prior to plunging in liquid ethane.

Data were collected on a Glacios (TFS) with XFEG operating at 200KeV with a selectris energy filter and falcon 4i camera. Data were collected with a pixel size of 0.92 Angstrom per pixel in .EER format over a targeted defocus range of −0.7um to −2.0 um with a target dose of 30 electrons per square Angstrom, using EPU.

All movies were motion corrected in cryoSPARC^33^ using patch motion correction and binned by ½ to a pixel size of 1.84 Angstrom per pixel. Motion corrected micrographs were CTF corrected using patch CTF in cryoSPARC. All downstream processing was performed in cryoSPARC v4.6.2^34^.

### 5mC-6HBC data processing

999 micrographs were denoised and used for blob picking. 38,981 particles were extracted with a box size of 360px downsampled to 180px and subjected to 3 rounds of 2D classification, resulting in 20,062 selected particles. These were used for 3 class ab initio reconstruction and the best class contained 8413 particles, which were used for topaz training for repicking the 999 micrographs. After Topaz^35^ picking, 24,803 particles were extracted with a 360 pixel box size downsampled to 180 pixels. These were put through 2 rounds of 2D classification to remove bad picks resulting in 17,826 particles that were used for a 3-class ab initio reconstruction. From this emerged a monomer class, a type-1 dimer class and a small class.

The dimer class contained 4441 particles and was further sorted in 3D by another 2 class ab initio reconstruction job, yielding 2616 particles that make up the final dimer particle stack. These were refined with homogenious refinement, followed by local refinement with C1 symmetry to reach 12.1 Angstrom GSFSC (0.143). Local refinement with C2 symmetry only improved the GSFSC (0.143) marginally to 11.8 Angstrom for the 5mC-6HBC-T1-dimer map.

The monomer class contained 7648 particles which were re-extracted with a box size of 256 px without down sampling. 1-class ab initio followed by homogeneous refinement and then local refinement refinement reached a GS-FSC (0.143) of 7.87 Angstrom for the monomer 5mC-6HBC-monomer map.

### 5mC-TC-6HBC data processing

4,417 micrographs were used for blob picking resulting in 443,336 extracted particles with a box size of 256 pixels. 2D classification to remove junk particles left 387,476 particles. 3-class ab initio reconstruction with 40,000 particles followed by heterogeneous refinement with all 387,476 particles resulted in 2 junk classes and 1 class with 5 visible helices and a fuzzy density where the sixth helix should be. 2-class ab initio with the 144,174 particles from the best class was performed, followed by non-uniform refinement with the 96,529 particles from the best class. These 96529 particles were used for 3D classification without alignment into 5 classes and identified 1 class with an intact sixth helix. The 19,587 particles from this class were re-extracted with a box size of 256 without further downsampling and we performed a final ab initio reconstruction, homogeneous refinement and then local refinement to arrive at the mature 5mC-TC-6HBC-monomer map at 7.95 Angstrom GSFSC (0.143) resolution.

A separate workflow was used to classify the dimer particles, beginning with 583,434 particles from blob picking, 2 rounds of 2D classification were performed to remove junk. 5-class ab initio reconstruction with the remaining 293,927 particles resulted in 1 class that had 5 clear helixes and an additional helix nearby but not attached. 3-class ab initio with the 73,602 particles from that class was performed followed by a 2-class ab initio with the 21,466 particles from that best class. Each of these two classes had partially resolved second units. 11,203 particles from the better class were re-extracted with a box size of 360 px and ab initio reconstruction followed by homogeneous refinement and then local refinement was performed to arrive at the best type-2 5mC-TC-6HBC map with GSFSC (0.143) resolution of 11.3.

### N1Ψ-TC-6HBC data processing

3583 micrographs were denoised and used for blob picking, 257,097 particles were extracted with a box size of 256 pixels and downsampled to 128 pixels. 2D classification removed 30,106 junk particles. The remaining 221,726 particles were used for a 5 class ab initio reconstruction and the best class (64,995 particles) were then used for a 4 class ab initio reconstruction. The best 3 classes (59,647 particles) were then used for a 5 class ab initio reconstruction, resulting in 4 classes resembling the type 2 dimer and 1 class resembling the type 1 dimer. The best type 2 dimer class contained 5,904 particles, which were re-extracted with a box size of 360 pixels. These were used for ab initio reconstruction followed by homogeneous refinement resulting in a GSFSC (0.143) of 12.12 Angstrom. The type-1 dimer 8,333 particles were re-centered and re-extracted with a box size of 360 pixels and ab intio reconstruction followed by homogeneous refinement resulting in a GSFSC (0.143) of 11.5 Angstrom

### 5mC-TR data processing

5214 micrographs were denoised and used for blob picking. 1,388,844 particles were extracted with a box size of 196 pixels and downsampled to 98 pixels. 2-class ab initio resulted in 1 junk and 1 good class. 2 copies of the good class and the junk class were then used for 3 class heterogeneous refinement with the particles (599,317) from the two good classes. This resulted in 233,268 particles in the better resolving good class. These particles were re-extracted with a box size of 196 and no downsampling and homogeneous refinement followed by non uniform refinement resulted in a map with a GSFSC of 4.85 Angstrom for the 5mC-TR map.

### Mass photometry

Mass photometry measurements were performed at 22°C using a Refeyn TwoMP system (Refeyn Ltd). Silicone gaskets and glass coverslips (MP-con-21008) were purchased from Refeyn. The coverslips were coated with poly-L-lysine (Sigma P8920) by applying 7 μL of a 0.1% solution and sandwiching them crosswise with another coverslip. This resulted in the formation of a thin layer of poly-L-lysine solution between the coverslips. After 20 seconds of incubation, the coverslips were washed with Milli-Q water and air-dried. Sample chambers were assembled by placing a coverslip on top of a silicone gasket. The instrument lens was coated with immersion oil (Zeiss Immersol 518F), and the assembled sample chambers were placed on the MP sample stage.

18 μL of buffer (25 mM Hepes, pH 8.0, 50 mM KCl, 5 mM MgCl_2_) was added to the sample chamber, and the coverslip was focused. 2 μL of the RNA sample was added and mixed immediately prior to data acquisition. Data were acquired using Refeyn AcquireMP software over the course of 1 minute with a regular image size. Mass calibration was conducted using Millennium^™^ RNA Markers (ThermoFisher Scientific AM7150) as standards. Mass photometry data for the RNA sample and standards were analyzed using Refeyn DiscoverMP software.

### TR activity assays:

For the gel based assays a Cy5–5’-CCCUCUAAAAA-3’ RNA substrate was purchased from Integrated DNA technologies. Reactions were mixed with a final buffer composition of 25mM HEPES pH 8.0, 10mM MgCl2, 1mM GTP, 150nM substrate, 15mM TR and a total volume of 25uL. 50 degree refolded, N-state, TR was used unless otherwise mentioned. After mixing reactions were incubated in a thermal cycler at the indicated temperatures and times. Reactions were quenched with EDTA to a final concentration of 100mM and then an equal volume of formamide was added. Samples were heated for 3 minutes at 90C and then brought to 4 degrees. 4uL of sample was loaded onto a 10% acrylamide (29:1) TBE gel for electrophoresis. Gels were imaged on an Biorad ChemiDoc.

For the platereader based assays we followed the protocol developed by Potraz and Russell^35^ and ordered 6-FAM-5’-CCCUCUAAAAA-BHQ1 from Integrated DNA technologies for the substrate. These reactions were performed with 25mM HEPES pH 8.0, 10mM MgCl2, 1mM GTP, 1000nM substrate, 15mM TR. Samples were prepared in a 384 well plate, 2uL of substrate was added last to the 38uL of reaction mixture followed by mixing by vigorous shaking in the plate reader with an estimated 15 second delay from substrate addition to first read. Samples were excited with 490nm light and emission was measured at 530nm. Readings were paused briefly to set the temperature on the plate reader and then resumed for subsequent readings.

### TR FRET assay

For the double labelling of TR we followed the protocol by Xiao et al^36^. For the first label 100pmol of RNA was mixed with 200pmol of each oligo from Supplemental table 2 with the A125 or SSb prefix were annealed in a thermal cycler. NAI-N3 treatment was performed as described, followed by purification and copper-free click reaction with Az-488-DBCO. For the second label, oligoes from Supplemental Table 2 with the prefix A325 or SSb were annealed to the RNA. The second NAI-N3 treatment, purification, and coupling to TAMRA-DBCO was performed followed by further DNase treatment and a final purification by RNA cleanup column (NEB) and eluted in 50mM HEPES pH 8.0 and diluted to 5uM. Samples were refolded by heating to 90C for 3 minutes and cooling at 25C for 15 minutes, followed by the addition of MgCl2 to a final concentration of 10mM and a 15-minute incubation at 25C. The samples were then incubated for 30 minutes at 50C and returned to room temperature.

Samples were diluted to 250nm and 50uL was added to a black 384 well plate (corning) for fluorescence measurements. Measurements were taken on an iD5 (molecular devices) plate reader with excitation at 485 nm and monitoring emission from 525–650nm in 2 or 5nm wavelength increments, as well as excitation at 550 and emission from 590–650. Ambient temperature was recording during each reading and 30 minutes was allowed for the samples to equilibrate temperature once the ambient temperature reached either 42 or 27C. The same samples were measured across 3 heating and cooling cycles. The emission spectra from the 485nm excitation were normalized to the emission spectra from 550nm excitation, emission at 590nm (direct acceptor fluorescence, values for each sample to control for labelling efficiency. The spectra were then normalized to at 535nm. A singly labelled (donor only, Az-488) RNA was used as a negative control.
